# 6,7-Dihydroxy-4-methylcoumarin Suppresses Adipogenesis via AMPK and MAPK Signaling with In Silico Analysis of Adipogenic Proteins

**DOI:** 10.3390/ph18121780

**Published:** 2025-11-23

**Authors:** Ye-Jin Lee, Yang Xu, Chang-Gu Hyun

**Affiliations:** Jeju Inside Agency and Cosmetic Science Center, Department of Chemistry and Cosmetics, Jeju National University, Jeju 63243, Republic of Korea; yyyyejin615@gmail.com (Y.-J.L.); iamxuyang1990@gmail.com (Y.X.)

**Keywords:** 3T3-L1 cells, 4-methylcoumarin, AMPK activation, anti-obesity agent, lipid accumulation, MAPK signaling, molecular docking, molecular dynamics simulations

## Abstract

**Background/Objectives**: Coumarin-based compounds exhibit diverse pharmacological properties, and 4-methylcoumarin (4MC) has emerged as a promising scaffold for drug development. However, its anti-obesity mechanisms remain insufficiently understood. This study aimed to evaluate the anti-adipogenic potential of 4MC derivatives in 3T3-L1 preadipocytes and to elucidate their underlying molecular mechanisms. **Methods**: 3T3-L1 preadipocytes were treated with structurally diverse 4MC derivatives. Lipid accumulation was analyzed using Oil Red O staining, cell viability by MTT assay, and the expression of adipogenic proteins by Western blotting. Molecular docking and molecular dynamics simulations were performed to predict the interactions between lead compounds and key adipogenic regulators. **Results:** Among the tested derivatives, 6,7-dihydroxy-4-methylcoumarin (6,7DH-4MC) markedly inhibited lipid accumulation in a dose-dependent manner without cytotoxicity. It suppressed the expression of major adipogenic transcription factors (PPAR-γ, C/EBPα, SREBP-1c) and FABP4. Additionally, 6,7DH-4MC inhibited ERK1/2 and p38 MAPK phosphorylation while activating AMPK. It also reduced CREB phosphorylation, indicating suppression of early adipogenesis. Computational analyses revealed stable binding of 6,7DH-4MC within the active sites of multiple adipogenic regulators, supporting its pleiotropic mode of action. **Conclusions:** 6,7DH-4MC exerts potent anti-adipogenic effects by modulating key adipogenic signaling pathways and transcriptional networks. These findings highlight 6,7DH-4MC as a promising lead compound for anti-obesity drug development, warranting further in vivo studies.

## 1. Introduction

Obesity has emerged as a significant global clinical challenge in modern society, necessitating an in-depth understanding of its multifaceted impact. The health consequences of obesity are profound, as it substantially increases the risk of various chronic diseases, including type 2 diabetes, hypertension, cardiovascular diseases, non-alcoholic fatty liver disease (NAFLD), and stroke. Notably, abdominal obesity is a key contributor to metabolic syndrome, characterized by abnormalities in blood glucose, lipid levels, and blood pressure, which collectively lead to a rapid rise in metabolic syndrome cases. Furthermore, obesity is recognized as a major risk factor for the development of certain cancers, such as colorectal and breast cancer [1,2,3].

Beyond its health implications, obesity poses significant social and economic burdens. The management and treatment of obesity-related diseases incur substantial healthcare costs, contributing to an increased economic strain on society. Additionally, obesity negatively impacts mental health, increasing the prevalence of anxiety and depression, and ultimately diminishes quality of life. Physical discomfort and chronic fatigue associated with obesity also lead to reduced productivity in professional and social settings. Childhood obesity, in particular, is linked to a higher likelihood of persisting into adulthood, thereby elevating the risk of early-onset chronic diseases [3,4,5,6].

To address the obesity epidemic, a multifaceted approach encompassing pharmacological treatment, dietary control, and lifestyle modifications is essential. However, the long-term use of anti-obesity drugs is often limited due to adverse effects, including rebound weight gain and increased cardiovascular risks. For instance, orlistat, a lipase inhibitor, is effective in reducing fat absorption but is associated with side effects such as steatorrhea, gastrointestinal discomfort, and fat-soluble vitamin deficiencies. Lorcaserin, a serotonin receptor agonist, was withdrawn from the market due to concerns about various cancer risks and cardiovascular safety. Similarly, phentermine, a sympathomimetic agent, has been linked to insomnia, increased heart rate, and hypertension. More recently, GLP-1 receptor agonists such as semaglutide, despite their efficacy, have reported side effects including nausea, vomiting, and gallbladder-related issues [7,8,9,10,11,12,13].

Given these challenges, natural product-based anti-obesity agents have gained significant attention as safer and more effective alternatives. Natural compounds, derived from traditional medicinal plants and dietary sources, often exhibit superior safety profiles due to their long history of use. These bioactive compounds modulate multiple pathways, such as inhibiting adipocyte differentiation, promoting fatty acid oxidation, and suppressing inflammation, thereby contributing to their anti-obesity effects. Moreover, many natural products offer additional health benefits, including anti-inflammatory, antioxidant, and glucose-regulatory properties, making them valuable in comprehensive obesity management [14,15].

In this context, 3T3-L1 cells, derived from mouse embryonic fibroblasts, serve as a widely utilized in vitro model for investigating adipogenesis and lipid metabolism. These preadipocytes can be induced to differentiate into mature adipocytes through treatment with insulin, dexamethasone, and 3-isobutyl-1-methylxanthine (IBMX), closely mimicking the in vivo adipocyte differentiation process. Differentiated 3T3-L1 cells exhibit hallmark adipocyte characteristics, such as lipid droplet accumulation and triglyceride storage, which allow for the assessment of lipogenesis, lipolysis, and energy metabolism pathways. The robust and reproducible nature of this model makes it a powerful tool for anti-obesity compound screening, elucidation of metabolic disease mechanisms, and studies on inflammation and insulin signaling [16,17,18].

Structurally, 4-methylcoumarin (4MC) consists of a benzopyran-2-one (coumarin) core with a methyl group substituted at the 4-position. This substitution modifies the electronic distribution of the molecule and enhances its lipophilicity, which may influence membrane permeability and interaction with biological targets. The basic scaffold supports diverse chemical modifications, such as hydroxylation or methoxylation, allowing for the generation of a wide variety of derivatives with distinct bioactivities. 4MC derivatives are naturally found in various plant species such as Angelica archangelica (commonly known as garden angelica), Dipteryx odorata (tonka bean), and Citrus aurantium (bitter orange). These plants are known to biosynthesize coumarin-based secondary metabolites as part of their chemical defense systems or aroma profiles. 4MC exhibits potent antioxidant and free radical-scavenging activities, preventing oxidative stress-induced cellular damage [19,20]. Unlike conventional coumarins, 4MC is not metabolized by P450 enzymes into toxic epoxide intermediates, which enhances its safety profile [21]. Studies have demonstrated that 4MC and its derivatives suppress cell proliferation and induce apoptosis in various cancer cell lines, such as K562, LS180, and MCF-7, while also exerting anti-inflammatory effects by inhibiting the production of nitric oxide, thromboxane B2, prostaglandin E2 (PGE2), and TNF-α [22,23]. Additionally, 4MC displays antibacterial activity, highlighting its potential as an anti-tuberculosis agent, and neuroprotective properties that may benefit the treatment of Alzheimer’s and Parkinson’s diseases. Furthermore, 4MC acts as a platelet aggregation inhibitor and a thromboxane A2 receptor antagonist, providing a therapeutic option for patients resistant to conventional antiplatelet therapy [24,25,26,27]. Collectively, these findings position 4MC as a versatile compound with significant pharmacological potential.

**Figure 1:**
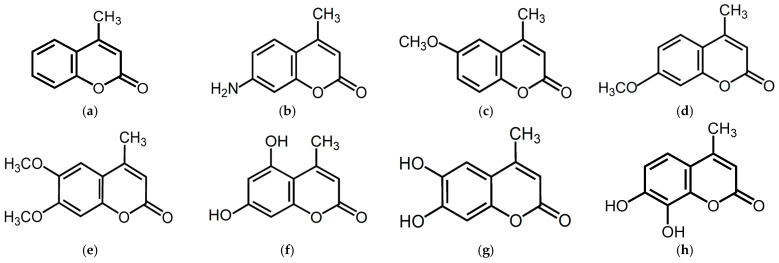
The chemical structures of (**a**) 4-methylcoumarin (4MC), (**b**) 7-amino-4-methylcoumarin (7A-4MC), (**c**) 6-methoxy-4-methylcoumarin (6M-4MC), (**d**) 7-methoxy-4-methylcoumarin (7M-4MC), (**e**) 6,7-dimethoxy-4-methylcoumarin (6,7DM-4MC), (**f**) 5,7-dihydroxy-4-methylcoumarin (5,7DH-4MC), (**g**) 6,7-dihydroxy-4-methylcoumarin (6,7DH-4MC), and (**h**) 7,8-dihydroxy-4-methylcoumarin (7,8DH-4MC).

Despite its diverse bioactivities, studies investigating the relationship between 4MC derivatives and anti-obesity effects remain limited. As part of our ongoing screening program to identify bioactive natural compounds with anti-inflammatory properties and potential effects on melanogenesis, we have identified several chalcones, sophorenins, and coumarins. In this study, we focused on 4MC and its structurally modified derivatives to explore their anti-obesity effects in 3T3-L1 adipocytes. Specifically, we examined the effects of seven 4MC derivatives, namely 6-methoxy-4-methylcoumarin (6M-4MC), 7-methoxy-4-methylcoumarin (7M-4MC), 7-amino-4-methylcoumarin (7A-4MC), 5,7-dihydroxy-4-methylcoumarin (5,7DH-4MC), 6,7-dihydroxy-4-methylcoumarin (6,7DH-4MC), 7,8-dihydroxy-4-methylcoumarin (7,8DH-4MC), and 6,7-dimethoxy-4-methylcoumarin (6,7DM-4MC), to elucidate their structural characteristics and potential anti-obesity effects (Figure 1). This research aims to provide valuable insights into the development of novel natural product-based anti-obesity agents.

## 2. Results

### 2.1. Cell Viability of 4MC Derivatives in 3T3-L1 Adipocyte Cells

**Figure 2:**
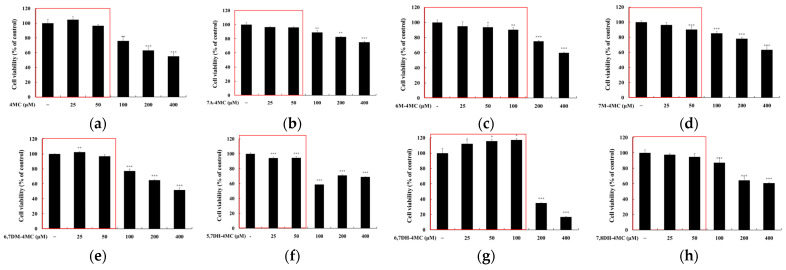
The effects of 4MC and its derivatives on the viability of 3T3-L1 adipocytes cells. The cells were treated with various concentrations (25–400 μM) of 4MC (**a**), 7A-4MC (**b**), 6M-4MC (**c**), 7M-4MC (**d**), 6,7DM-4MC (**e**), 5,7DH-4MC (**f**), 6,7DH-4MC (**g**), and 7,8DH-4MC (**h**) for 24 h. Cell viability was expressed as percentage relative to untreated cells. Red boxes indicate the concentration range used for subsequent adipogenesis experiments. Results are expressed as the mean ± SD of three replicate experiments. * *p* < 0.05, ** *p* < 0.01, *** *p* < 0.001.

To eliminate data errors during the investigation of the relationship between 4MC derivatives and anti-obesity effects, we assessed the potential cytotoxicity of the compounds in 3T3-L1 adipocytes by performing an MTT assay using concentrations ranging from 25 to 400 μM. The cells were incubated with each compound for 72 h, and cell viability was considered unaffected when the survival rate was 90% or higher compared to the untreated control group. At a concentration of 50 μM, most 4MC derivatives did not exhibit cytotoxicity. Specifically, 4MC, 7A-4MC, 6M-4MC, 7M-4MC, 6,7DM-4MC, 5,7DH-4MC, and 7,8DH-4MC maintained cell viability above 90%, indicating minimal or no cytotoxic effects. Notably, 6,7DH-4MC showed a significant increase in cell viability, reaching 115.7%, suggesting a potential proliferative effect at this concentration. At 100 μM, a more varied response was observed. While compounds such as 6M-4MC maintained a cell viability rate of 90.4%, indicating negligible cytotoxicity, 4MC, 7A-4MC, 7M-4MC, and 6,7DM-4MC showed reduced survival rates, although these were still above 75%, indicating a mild cytotoxic effect. However, 5,7DH-4MC exhibited a substantial decrease in cell viability, with a survival rate of only 59.3%, indicating significant cytotoxicity at this concentration. Conversely, 6,7DH-4MC showed a modest increase in cell viability (117.3%) at 100 μM. However, this enhancement is likely within the range of metabolic activation rather than a true proliferative effect, as no morphological changes or growth-related alterations were observed. These results indicate that most 4MC derivatives exhibit low cytotoxicity at 50 μM, whereas higher concentrations may lead to variable cellular responses. To avoid unintended effects associated with elevated metabolic activity, all subsequent experiments were performed at concentrations of 50 μM or lower, where no cytotoxicity was detected (Figure 2).

### 2.2. The Effects of 4MC and Its Derivatives on Lipid Accumulation in 3T3-L1 Adipocytes

**Figure 3:**
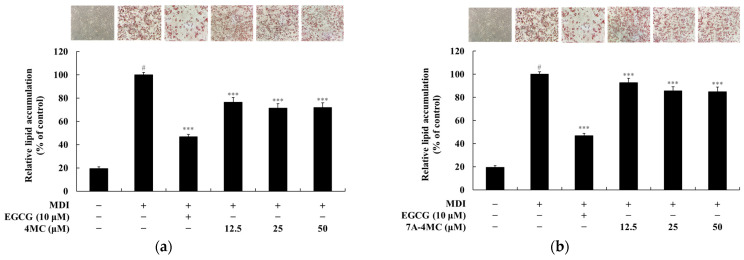

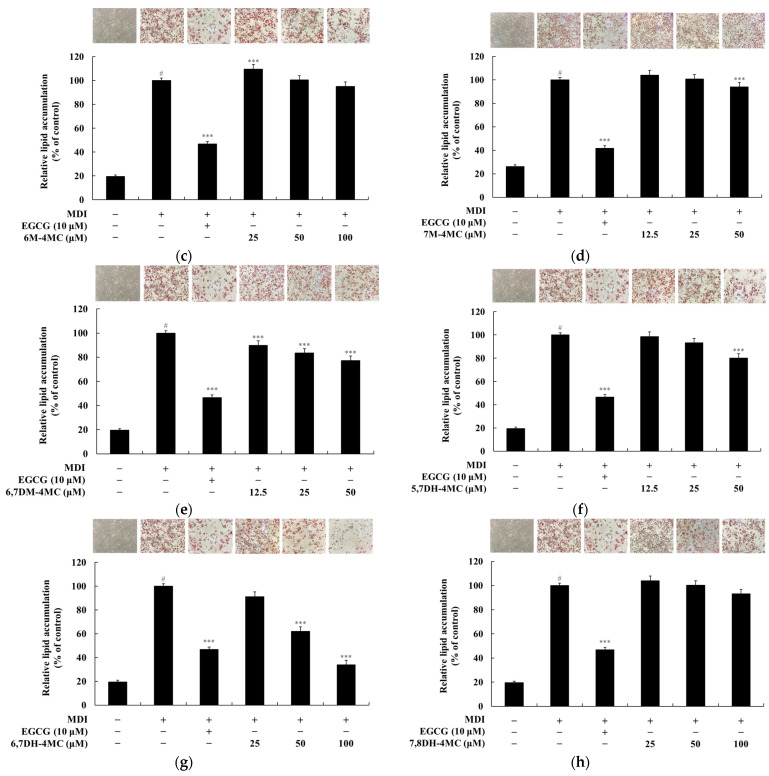
Effect of 4MC and its derivatives on lipid accumulation in 3T3-L1 adipocytes. Oil Red O staining was performed after 8 days of differentiation to visualize lipid droplets (red, ×100 magnification). Lipid accumulation was quantified and presented as a graph. 3T3-L1 cells were treated for 72 h with 4MC and its derivatives at different concentrations: 12.5, 25, and 50 μM for 4MC (**a**), 7A-4MC (**b**), 7M-4MC (**d**), 6,7M-4MC (**e**), and 5,7DH-4MC (**f**); and 25, 50, and 100 μM for 6M-4MC (**c**), 6,7DH-4MC (**g**), and 7,8DH-4MC (**h**). Note: Panel (**d**) was obtained from an experiment conducted on a different day; therefore, the MDI and EGCG control images in panel (**d**) differ from those used in the other panels. Data represent mean ± SD from three independent experiments. Statistical significance: *** *p* < 0.001 vs. MDI group; # *p* < 0.001 vs. untreated control.

To assess the lipid accumulation inhibitory effects of 4MC and its derivatives, we performed Oil Red O staining after 8 days of differentiation in 3T3-L1 adipocytes. This staining technique highlights intracellular triglycerides and neutral lipids in red, allowing for a visual and quantitative evaluation of lipid accumulation. The inhibitory potential of 4MC and its seven derivatives was analyzed by comparing the extent of lipid accumulation reduction relative to cells treated with the standard differentiation cocktail. As shown in Figure 3, treatment with the methylisobutylxan-thine-dexamethasone-insulin (MDI) cocktail led to more than a fivefold increase in adipocyte differentiation compared to the untreated control group, confirming successful induction of adipogenesis. To validate the experimental setup, epigallocatechin gallate (EGCG), a well-known inhibitor of lipid ac-cumulation derived from green tea, was used as a positive control and reduced lipid accumulation by 53.3% at 10 μM. EGCG has been widely reported to exert anti-obesity effects through multiple mechanisms, including the activation of AMP-activated protein kinase (AMPK), which leads to the inhibition of lipogenesis and promotion of fatty acid oxidation. Additionally, EGCG modulates adipogenic transcription factors such as PPAR-γ and C/EBPα, and suppresses the expression of genes involved in lipid uptake and triglyceride synthesis. A previous study by Peng et al. [28] further demonstrated that EGCG inhibits mitotic clonal expansion (MCE), an essential early step in adipocyte differentiation. This action results in the arrest of the cell cycle, leading to suppression of both early and terminal adipocyte differentiation and overall lipid accumulation. Based on these well-established mechanisms, EGCG was employed as a reliable positive control in this study to benchmark the lipid-lowering efficacy of 6,7DH-4MC [28,29]. The effects of 4MC and its derivatives on lipid accumulation varied depending on the compound and concentration. Treatment with 4MC at 12.5, 25, and 50 μM resulted in inhibition rates of 23.6%, 28.6%, and 28.0%, respectively. 7A-4MC demonstrated weaker effects, with inhibition rates of 7.4%, 14.5%, and 15.0% at the same concentrations. 6M-4MC showed minimal inhibition, achieving only a 5.0% reduction at 100 μM. For 6,7M-4MC, the inhibition rates were 10.3%, 16.6%, and 23.0% at 12.5, 25, and 50 μM, respectively. Treatment with 7M-4MC at 50 μM resulted in a minimal inhibition rate of 6.0%. Similarly, 7,8DH-4MC exhibited minimal inhibitory effects, achieving only a 6.0% reduction at 100 μM. Interestingly, 5,7DH-4MC exhibited only minor inhibition at lower concentrations, with inhibition rates of 1.4% and 6.7% at 12.5 and 25 μM, but the rate reached 20.0% at 50 μM. In contrast, 6,7DH-4MC displayed robust dose-dependent inhibition, with rates of 8.9%, 38.0%, and 66.2% at 25, 50, and 100 μM, respectively. Overall, these findings indicate that while the lipid accumulation inhibitory effects of most 4MC derivatives increased with higher concentrations, significant differences were observed among the derivatives. Notably, 6,7DH-4MC exhibited the strongest inhibitory effect on lipid accumulation, leading to further mechanistic studies to gain deeper insights into its anti-adipogenic properties.

### 2.3. The Effects of 6,7DH-4MC on Marker Protein Expression in 3T3-L1 Adipocytes

**Figure 4:**
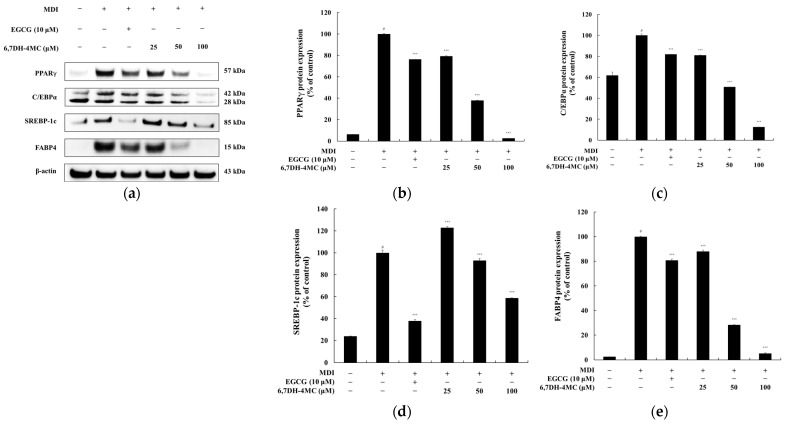
Effects of 6,7DH-4MC on the expression of adipocyte differentiation marker proteins in 3T3-L1 Cells. This figure illustrates the effects of 6,7DH-4MC on the expression of key adipocyte differentiation marker proteins in MDI-induced 3T3-L1 adipocytes. Panel (**a**) presents representative Western blot images showing the protein levels of PPAR-γ (57 kDa), C/EBPα (42 kDa and 28 kDa), SREBP-1c (85 kDa), and FABP4 (15 kDa) following treatment with MDI alone, EGCG (10 μM), or 6,7DH-4MC at various concentrations (25, 50, and 100 μM). β-Actin (43 kDa) was used as a loading control to ensure equal protein loading across samples. The bar graphs in panels (**b**–**e**) provide a quantitative analysis of the protein expression levels, normalized to β-actin and expressed as a percentage of the control group. Specifically, the bar graphs show (**b**) PPAR-γ, (**c**) C/EBPα, (**d**) SREBP-1c, and (**e**) FABP4 expression. Statistical analysis indicates significant differences compared to the control and MDI-treated groups (*** *p* < 0.001). # *p* < 0.001 vs. untreated control.

To investigate the anti-adipogenic effects of 6,7DH-4MC, we analyzed the expression of adipocyte differentiation marker proteins, including peroxisome proliferator-activated receptor gamma (PPAR-γ), CCAAT/enhancer binding protein alpha (C/EBPα), sterol regulatory element-binding protein 1c (SREBP-1c), and fatty acid-binding protein 4 (FABP4), in 3T3-L1 preadipocytes [30]. The results show that upon treatment with MDI (a mixture used to induce adipocyte differentiation), the expression of PPAR-γ increased 15.82-fold, while the positive control EGCG (10 μM) exhibited an inhibitory effect of 23.7%. In comparison, 6,7DH-4MC showed inhibitory effects of 20.6%, 62.1%, and 97.4% at 25, 50, and 100 μM, respectively. In the case of C/EBPα, MDI treatment resulted in a 1.67-fold in-crease, and EGCG (10 μM) inhibited its expression by 18.1%. 6,7DH-4MC treatment at 25, 50, and 100 μM led to inhibition rates of 19.91, 49.2%, and 81.3%, respectively. For SREBP-1c, MDI treatment led to a 4.18-fold increase in expression, while EGCG (10 μM) inhibited it by 62.2%. 6,7DH-4MC treatment resulted in inhibition rates of 7.0% at 50 μM and 41.3% at 100 μM. Regarding FABP4, MDI treatment increased its expression 37.74-fold, while EGCG (10 μM) showed an inhibitory effect of 19.3%. 6,7DH-4MC exhibited inhibitory effects of 12.1%, 71.8%, and 94.7% at 25, 50, and 100 μM, respectively (Figure 4). These findings demonstrate that 6,7DH-4MC effectively suppresses adipocyte differentiation and lipid accumulation by inhibiting the expression of key adipocyte differentiation marker proteins, including PPAR-γ, C/EBPα, SREBP-1c, and FABP4. The particularly high inhibition rates of FABP4 and PPAR-γ suggest that 6,7DH-4MC plays a significant role in suppressing lipid droplet formation during the late stages of adipocyte differentiation. In conclusion, 6,7DH-4MC exerts anti-adipogenic effects by effectively inhibiting the expression of major marker proteins involved in adipocyte differentiation and lipid metabolism. This study highlights the potential of 6,7DH-4MC as a promising candidate for further development as an anti-obesity agent.

### 2.4. The Effect of 6,7DH-4MC on AKT Phosphorylation in 3T3-L1 Adipocytes

**Figure 5:**
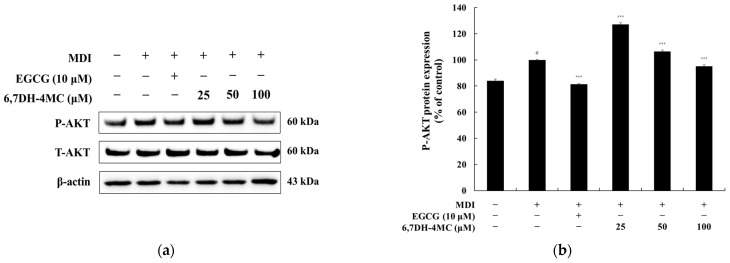
The effects of 6,7DH-4MC on AKT phosphorylation in MDI-induced 3T3-L1 adipocytes. Panel (**a**) shows representative Western blot images of phosphorylated AKT (P-AKT, 60 kDa) and total AKT (T-AKT, 60 kDa) in untreated, MDI-treated, EGCG-treated (10 μM), and 6,7DH-4MC-treated groups (25, 50, and 100 μM). Panel (**b**) presents the quantification of P-AKT expression normalized to T-AKT levels and expressed as a percentage of the control group. The relative intensity of the protein bands was quantified using ImageJ software version 9.4.0, and the values were normalized to the corresponding loading control. Untreated cells were considered as 100%. Data are expressed as the mean ± SD of three independent experiments (^#^ *p* < 0.001 compared to the untreated control; *** *p* < 0.001 indicate significant differences compared to the MDI group.

AKT is a central protein in the PI3K/AKT signaling pathway, which plays a crucial role in regulating cell survival, growth, and metabolism. Notably, it regulates various functions related to adipocyte differentiation (adipogenesis). The activation of AKT is controlled through phosphorylation, significantly influencing adipocyte differentiation and lipid metabolism [31]. To investigate the relationship between the adipogenesis-inhibitory effects of 6,7DH-4MC and the suppression of marker protein expression with AKT activation, we analyzed the phosphorylation levels of AKT. Figure 5 presents the phosphorylation levels of AKT (Ser473) relative to total AKT protein expression. The results show that MDI treatment increased AKT phosphorylation 1.19-fold, while EGCG (10 μM) inhibited this increase by 18.7%. In contrast, 6,7DH-4MC at the highest concentration of 100 μM exhibited a modest inhibition of 4.9% (Figure 5). These findings suggest that 6,7DH-4MC exerts its anti-adipogenic effects through mechanisms that may be independent of strong AKT phosphorylation inhibition, warranting further investigation into additional regulatory pathways.

### 2.5. The Effect of 6,7DH-4MC on CREB Phosphorylation in 3T3-L1 Adipocytes

**Figure 6:**
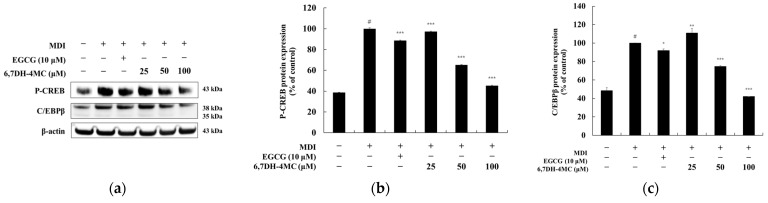
The effect of 6,7DH-4MC on P-CREB and C/EBPβ Expression in MDI-induced 3T3-L1 adipocytes. The expression levels of P-CREB and C/EBPβ in MDI-induced 3T3-L1 adipocytes treated with EGCG (10 μM) or 6,7DH-4MC (25, 50, 100 μM) were analyzed by Western blotting. (**a**) Western blot images showing phosphorylated CREB (P-CREB, 43 kDa) and C/EBPβ (38 kDa and 35 kDa) expression, with β-actin (43 kDa) as a loading control. (**b**) Quantification of P-CREB expression. MDI treatment significantly increased P-CREB levels (# *p* < 0.001 compared to untreated control), while EGCG and 6,7DH-4MC treatments reduced P-CREB levels in a dose-dependent manner. (**c**) Quantification of C/EBPβ expression. MDI treatment led to a significant increase (# *p* < 0.001), and 6,7DH-4MC treatment resulted in a marked reduction, especially at 100 μM. Protein band intensities were quantified using ImageJ software and normalized to β-actin. Data represent the mean ± SD from three independent experiments, with significance indicated as follows: * *p* < 0.05, ** *p* < 0.01, *** *p* < 0.001 compared to the MDI-treated group.

To investigate the relationship between the anti-adipogenic effects of 6,7DH-4MC and the regulation of P-CREB expression, we analyzed the phosphorylation levels of P-CREB in MDI-induced 3T3-L1 adipocytes (Figure 6). The phosphorylation level of P-CREB significantly increased 2.58-fold following MDI treatment, indicating enhanced activation of this transcription factor during adipogenesis. Treatment with EGCG (10 μM) reduced P-CREB phosphorylation by 11.4%. In contrast, 6,7DH-4MC exhibited a dose-dependent inhibitory effect, suppressing P-CREB phosphorylation by 2.8%, 34.8%, and 54.8% at concentrations of 25, 50, and 100 μM, respectively. These findings suggest that 6,7DH-4MC suppresses adipocyte differentiation by inhibiting P-CREB phosphorylation, a key transcription factor in the early stages of adipogenesis. The strong dose-dependent inhibition observed at higher concentrations of 6,7DH-4MC indicates its potential to disrupt the transcriptional cascade essential for adipocyte differentiation and lipid accumulation.

### 2.6. The Effect of 6,7DH-4MC on MAPK Signaling in MDI-Induced 3T3-L1 Adipocytes

**Figure 7:**
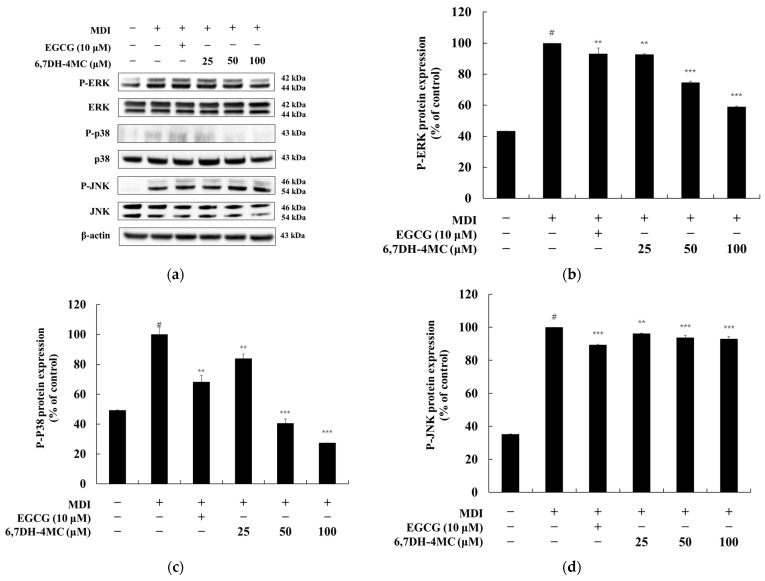
The Effect of 6,7DH-4MC on MAPK Phosphorylation in MDI-Induced 3T3-L1 Adipocytes. The expression levels of phosphorylated and total MAPK pathway proteins were analyzed by Western blotting in MDI-induced 3T3-L1 adipocytes treated with EGCG (10 μM) or 6,7DH-4MC (25, 50, 100 μM). (**a**) Western blot images of phosphorylated ERK1/2 (P-ERK), p38 MAPK (P-p38), and JNK (P-JNK), along with their respective total protein levels (ERK, p38, JNK). (**b**) Quantification of P-ERK/ERK expression. MDI significantly increased P-ERK levels (# *p* < 0.001), while EGCG and 6,7DH-4MC reduced these levels in a dose-dependent manner. (**c**) Quantification of P-p38/p38 ex-pression. P-p38 levels were markedly increased by MDI (# *p* < 0.001) and significantly reduced by 6,7DH-4MC, especially at 100 μM. (**d**) Quantification of P-JNK/JNK expression. MDI-induced P-JNK levels showed minimal inhibition by EGCG and 6,7DH-4MC. Protein band intensities were quantified using ImageJ and normalized to total protein levels. Untreated cells were set as 100%. Data represent mean ± SD from three independent experiments, with statistical significance indicated as follows: # *p* < 0.001 compared to the untreated control, ** *p* < 0.01, *** *p* < 0.001 compared to the MDI-treated group.

To investigate the relationship between the anti-adipogenic effects of 6,7DH-4MC and the MAPK signaling pathway [32], we analyzed the phosphorylation levels of extracellular signal-regulated kinases 1/2 (ERK1/2), p38 mitogen-activated protein kinase (p38 MAPK), and c-Jun N-terminal kinase (JNK) in MDI-induced 3T3-L1 adipocytes (Figure 7). ERK1/2 phosphorylation significantly increased 2.31-fold following MDI treatment, indicating enhanced activation during adipogenesis. Treatment with EGCG (10 μM) reduced ERK1/2 phosphorylation by 6.8%, while 6,7DH-4MC inhibited ERK1/2 phosphorylation by 7.4%, 25.4%, and 41.0% at concentrations of 25, 50, and 100 μM, respectively. p38 MAPK phosphorylation increased 2.02-fold following MDI treatment. EGCG (10 μM) reduced p38 MAPK phosphorylation by 31.7%, and 6,7DH-4MC showed a strong dose-dependent inhibition, with reductions of 16.1%, 59.4%, and 72.5% at 25, 50, and 100 μM, respectively. In contrast, JNK phosphorylation increased 2.83-fold with MDI treatment. However, EGCG (10 μM) only slightly inhibited JNK phosphorylation by 10.7%, and 6,7DH-4MC showed minimal effects, with inhibition levels of 3.7%, 6.2%, and 7.0% at 25, 50, and 100 μM, respectively. These findings suggest that 6,7DH-4MC exerts its anti-adipogenic effects primarily through the inhibition of ERK1/2 and p38 MAPK phosphorylation, while its impact on JNK phosphorylation remains relatively limited. The significant suppression of p38 MAPK phosphorylation, in particular, highlights a potential mechanism by which 6,7DH-4MC may regulate lipid metabolism and inhibit adipocyte differentiation.

### 2.7. The Effect of 6,7DH-4MC on AMPK Activation in MDI-Induced 3T3-L1 Adipocytes

**Figure 8:**
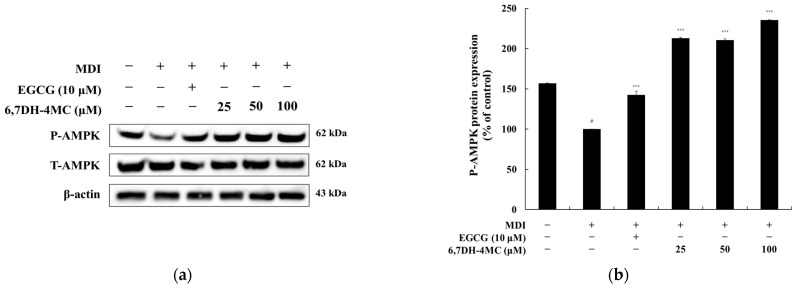
The effect of 6,7DH-4MC on AMPK activation in MDI-induced 3T3-L1 adipocytes. The expression levels of AMPK were analyzed by Western blotting to evaluate the phosphorylation of AMPK in MDI-induced 3T3-L1 adipocytes treated with EGCG (10 μM) or 6,7DH-4MC (25, 50, 100 μM). (**a**) Western blot images showing phosphorylated AMPK (P-AMPK, 62 kDa) and total AMPK (T-AMPK, 62 kDa), with β-actin used as a loading control. (**b**) Quantification of P-AMPK/T-AMPK expression. MDI treatment significantly reduced P-AMPK levels (# *p* < 0.001 compared to the un-treated control), indicating suppression of AMPK activation during adipocyte differentiation. EGCG treatment increased P-AMPK levels by activating AMPK. 6,7DH-4MC treatment led to a significant dose-dependent increase in P-AMPK levels, with the greatest effect observed at 100 μM. The relative intensities of the protein bands were quantified using ImageJ software and normalized to total AMPK levels. Untreated cells were set as 100%. Data represent the mean ± SD of three independent experiments, with statistical significance indicated as follows: # *p* < 0.001 compared to the untreated control, *** *p* < 0.001 compared to the MDI-treated group.

To investigate the relationship between the anti-adipogenic effects of 6,7DH-4MC and the AMPK signaling pathway, we analyzed the phosphorylation levels of AMPK in MDI-induced 3T3-L1 adipocytes (Figure 8). MDI treatment reduced AMPK phosphorylation by 35.9%, indicating a suppression of AMPK activity during adipocyte differentiation. In contrast, treatment with EGCG (10 μM) increased AMPK phosphorylation by 42.6%, serving as a positive control for AMPK activation. Notably, 6,7DH-4MC treatment resulted in a significant dose-dependent increase in AMPK phosphorylation, with 112.7%, 110.4%, and 135.6% increases at concentrations of 25, 50, and 100 μM, respectively. These findings indicate that 6,7DH-4MC effectively activates AMPK signaling, counteracting the MDI-induced suppression of AMPK phosphorylation. The substantial increase in AMPK activity suggests that 6,7DH-4MC may exert its anti-adipogenic effects by enhancing the AMPK-mediated inhibition of lipid synthesis and promoting fatty acid oxidation. This supports the hypothesis that AMPK activation plays a key role in the inhibitory effects of 6,7DH-4MC on adipocyte differentiation.

### 2.8. Pharmacokinetic and Drug-likeness Evaluation

The predicted absorption, distribution, metabolism, and excretion (ADME) profiles of 6,7DH-4MC and EGCG indicate that 6,7DH-4MC exhibits a favorable overall pharmacokinetic and drug-likeness profile for anti-obesity drug development (Table 1). Notably, 6,7DH-4MC shows high human intestinal absorption (93.81%), substantially higher than EGCG (57.66%), which may be advantageous for oral anti-obesity therapeutics. Although its Caco-2 permeability (−4.63 cm/s) is lower than that of EGCG (−0.66 cm/s), the overall higher absorption combined with the lack of P-gp inhibition (EGCG is a P-gp inhibitor) suggests improved oral bioavailability and a lower risk of transporter-mediated drug–drug interactions [33].

In terms of distribution, 6,7DH-4MC exhibits a higher fraction unbound (0.391 vs. 0.223 for EGCG), facilitating access to peripheral metabolic tissues such as the liver and adipose tissue. Its plasma protein binding is 72.1% (vs. 87.3% for EGCG), indicating a higher fraction of free drug (approximately 28% vs. 13%), which supports activity in peripheral tissues while reducing the risk of protein-binding related drug–drug interactions. Moreover, its BBB penetration index (−0.009 vs. −1.786 for EGCG) suggests minimal central nervous system exposure and a more predictable distribution in peripheral tissues, potentially supporting effective targeting of peripheral metabolic sites.

Regarding metabolism, 6,7DH-4MC inhibits only CYP1A2, whereas EGCG inhibits CYP3A4, the primary enzyme responsible for metabolizing over half of clinically used drugs [34]. This suggests that 6,7DH-4MC may have a lower risk of clinically significant drug–drug interactions.

For elimination, 6,7DH-4MC exhibits a moderate clearance (0.67 mL/min/kg) and a longer predicted half-life (1.662 h) compared with EGCG (T_1/2_ − 0.11 h), which may support sustained systemic exposure for potential long-term anti-obesity treatment.

Drug-likeness evaluation further underscores its advantage: 6,7DH-4MC is predicted to comply with Lipinski, Ghose, Veber, and Egan rules (Table 2), whereas EGCG violates multiple criteria due to high polarity (TPSA 197.4 Å^2^) and excessive numbers of hydrogen-bond donors and acceptors (HBD = 8, HBA = 11), resulting in limited oral permeability and suboptimal drug-like properties. In summary, the combination of high absorption, well-controlled peripheral distribution, minimal metabolic interaction risk, balanced clearance, and fully compliant drug-likeness suggests that 6,7DH-4MC could be a promising orally administered anti-obesity candidate.

### 2.9. Molecular Docking Simulations

This study revealed that 6,7-DH-4MC activates AMPK while suppressing the phosphorylation of ERK1/2, p38 MAPK, and CREB, thereby downregulating the transcription factor PPAR-γ and reducing the expression of its downstream effector gene FABP4. Based on these findings, ERK1/2 (PDB ID: 5LCJ), p38 (PDB ID: 1A9U), CREB (PDB ID: 4WHU), PPAR-γ (PDB ID: 2PRG), and FABP4 (PDB ID: 3FR2) were selected as molecular docking targets. In contrast, the transcription factors C/EBPα and SREBP-1c primarily regulate gene expression through DNA binding and protein–protein interactions. They are more likely to be modulated indirectly through upstream signaling pathways due to the lack of well-defined small-molecule binding pockets [35,36].

**Figure 9:**
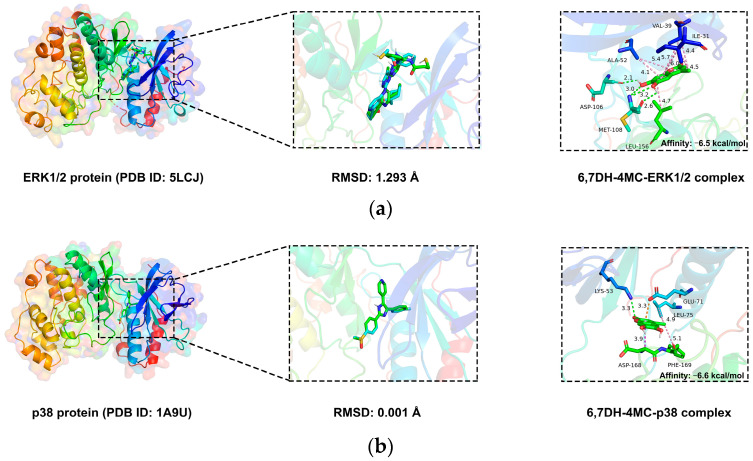

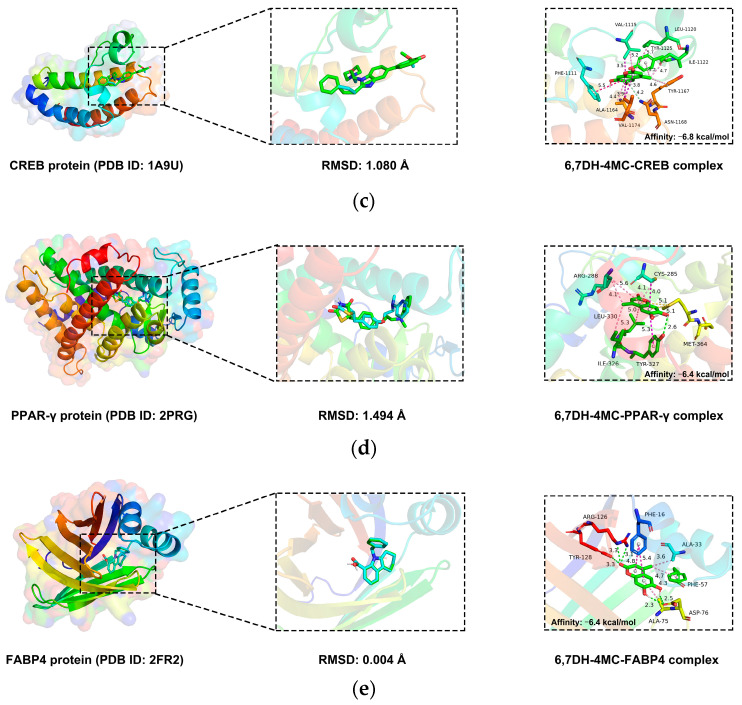
Molecular docking results of 6,7-DH-4MC with five target proteins: (**a**) ERK1/2, (**b**) p38, (**c**) CREB, (**d**) PPAR-γ, and (**e**) FABP4.

Prior to docking analysis, validation of the docking protocol confirmed that the RMSD values between pre- (green) and post-docking (blue) co-crystallized ligand conformations were below 2 Å, indicating reliable docking accuracy (Figure 9a–e). The calculated binding energies of 6,7-DH-4MC with ERK1/2, p38, CREB, PPAR-γ, and FABP4 were −6.5, −6.6, −6.8, −6.4, and −6.4 kcal/mol, respectively. Binding interaction analysis revealed that 6,7-DH-4MC formed multiple hydrogen-bonding and hydrophobic interactions with each target protein (Table 3). In the ERK1/2 complex, four hydrogen bonds were formed with ASP106 and MET108, while ILE31, VAL39, ALA52, and LEU156 contributed to hydrophobic stabilization. In the p38 complex, LYS53 participated in hydrogen bonding, whereas GLU71, LEU75, ASP168, and PHE169 were involved in hydrophobic interactions. The CREB complex was primarily stabilized by hydrophobic interactions with PHE1111, VAL1115, LEU1120, ILE1122, ALA1164, TYR1167, ASN1168, and VAL1174. In the PPAR-γ complex, TYR327 formed a hydrogen bond, while ARG288, CYS285, ILE326, TYR327, LEU330, and MET364 contributed to hydrophobic interactions. In the FABP4 complex, ASP76, ARG126, and TYR128 established five hydrogen bonds, and PHE16, ALA33, PHE57, and ALA75 engaged in hydrophobic interactions. Collectively, these results demonstrate that 6,7-DH-4MC can stably bind to multiple target proteins, providing a molecular basis for its multi-target inhibitory effects on adipogenesis.

### 2.10. Molecular Dynamics Simulations

**Figure 10:**
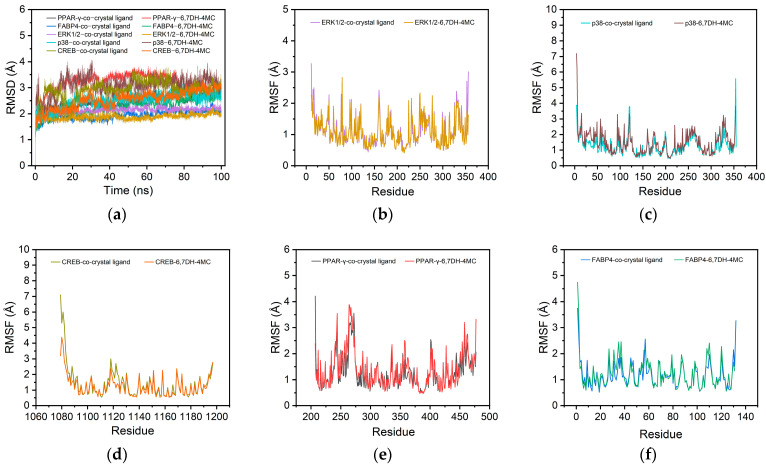
RMSD profiles of 6,7-DH-4MC–protein complexes (**a**). RMSF profiles of 6,7-DH-4MC–protein complexes: (**b**) ERK1/2, (**c**) p38, (**d**) CREB, (**e**) PPAR-γ, and (**f**) FABP4.

To further validate the molecular docking results, molecular dynamics (MD) simulations were conducted for 100 ns on the 6,7DH-4MC–protein complexes. The RMSD profiles showed minor structural fluctuations across all complexes, with trajectories remaining stable within 1.5–4 Å throughout the simulation period (Figure 10a), indicating that each complex exhibited overall structural stability. The RMSF analysis revealed that the profiles of the complexes with either the co-crystal ligand or 6,7-DH-4MC were largely overlapping, with fluctuations below 10 Å, indicating that 6,7-DH-4MC binding did not induce significant structural changes in the proteins and remained stably bound within the active pockets (Figure 10b–f).

Based on the equilibrated MD trajectories, binding free energies were calculated using the MM/GBSA method (Table 4). All complexes exhibited negative total binding free energies, implying spontaneous binding and the formation of thermodynamically stable complexes. Among them, ERK1/2 (−23.05 kcal/mol) and PPAR-γ (−23.00 kcal/mol) showed the strongest affinities toward 6,7-DH-4MC, followed by CREB (−20.45 kcal/mol), p38 (−17.90 kcal/mol), and FABP4 (−16.15 kcal/mol). Energy component analysis indicated that van der Waals interactions contributed most significantly to the overall binding stability, particularly in the ERK1/2 and PPAR-γ complexes, highlighting the dominant role of hydrophobic contacts in ligand binding.

The MM/GBSA residue energy decomposition further revealed that hydrophobic and aromatic residues predominantly stabilize 6,7-DH-4MC binding across all complexes (Table 5). In the ERK1/2 complex, TYR36 (−2.72 kcal/mol) and ALA52 (−1.90 kcal/mol) contributed most significantly, underscoring the key role of hydrophobic forces in complex stabilization. In p38, PHE169 (−1.86 kcal/mol) and GLU71 (−1.76 kcal/mol) made notable contributions to ligand binding. In the CREB complex, the energetic contributions of individual residues were relatively uniform and modest (−0.61 to −1.51 kcal/mol), indicating a distributed but stable interaction network. In PPAR-γ, TYR327 (−1.50 kcal/mol) and CYS285 (−1.32 kcal/mol) were the major stabilizing residues, whereas PHE57 (−3.12 kcal/mol) made the strongest contribution in the FABP4 complex. Collectively, these findings indicate that the binding of 6,7-DH-4MC to its protein targets is primarily driven by hydrophobic and aromatic interactions.

## 3. Discussion

Coumarin-based compounds represent an important class of bioactive molecules with diverse pharmacological activities, including anti-inflammatory, anticancer, and antimicrobial effects. Their structural diversity, combined with advancements in synthetic organic chemistry, facilitates the development of optimized lead compounds. This synergy between natural product chemistry and synthetic methodologies plays a pivotal role in drug development, enabling the creation of therapeutics targeting multiple disease pathways. Among these compounds, 4-methylcoumarin (4MC), a naturally occurring molecule found in plants and fungi, has garnered significant attention due to its antioxidant, anti-inflammatory, antimicrobial, and anticancer properties. As a small coumarin molecule, 4MC has been consistently explored as a promising starting point for drug discovery [37,38,39,40,41,42].

However, despite extensive studies on the antioxidant, anti-inflammatory, anticancer, and antimicrobial properties of coumarins, their mechanistic roles in adipogenesis have remained largely unexplored. In particular, no previous study has systematically compared structurally related 4MC derivatives or clarified how specific functional groups influence anti-adipogenic activity. The present work extends earlier coumarin research by (i) providing the first structure–activity relationship (SAR) analysis across seven 4MC derivatives, (ii) identifying 6,7-dihydroxyl substitution as a key pharmacophore essential for potent adipogenesis inhibition, and (iii) demonstrating a multi-target mechanism involving AMPK activation and concurrent suppression of ERK1/2, p38, and CREB signaling. Furthermore, the integrated use of molecular docking and MD simulations provides the first molecular-level evidence supporting direct multi-target interactions of a coumarin derivative in adipogenic pathways. These findings collectively position 6,7DH-4MC as a unique coumarin scaffold with mechanistic features not reported in prior studies.

**Figure 11:**
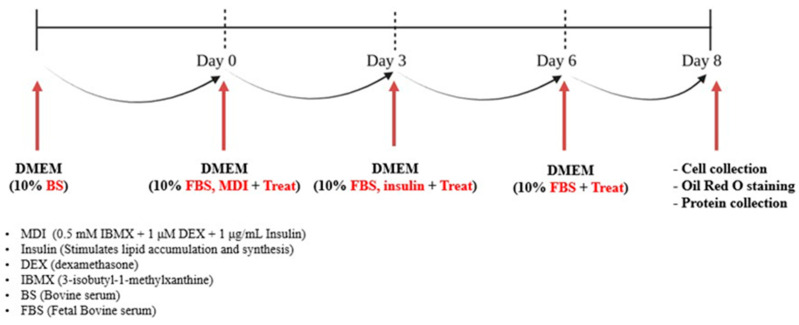
Overview of the experimental procedure and the timeline of adipogenesis in 3T3-L1 preadipocytes. On Day 0, cells are induced with DMEM containing 10% FBS, MDI (0.5 mM IBMX, 1 μM DEX, and 1 μg/mL insulin), and treatment compounds. On Day 3, the medium is replaced with DMEM containing 10% FBS, insulin, and treatment compounds. From Day 6 onwards, cells are maintained in DMEM containing 10% FBS and treatment compounds. On Day 8, cells are harvested for subsequent analyses, including Oil Red O staining for lipid accumulation, protein collection, and additional cellular assays.

In this study, the anti-adipogenic effects of 4MC and its derivatives (7A-4MC, 6M-4MC, 7M-4MC, 6,7DM-4MC, 5,7DH-4MC, 6,7DH-4MC, and 7,8DH-4MC) were evaluated by measuring lipid accumulation using Oil Red O staining after 8 days of differentiation in 3T3-L1 adipocytes (Figure 11). The MDI cocktail-treated control group exhibited a more than fivefold increase in adipocyte differentiation, whereas EGCG (10 μM) reduced lipid accumulation by 53.3%. 4MC showed inhibition rates of 23.6%, 28.6%, and 28.0%, depending on the concentration, while 6,7DH-4MC demonstrated dose-dependent inhibitory effects of 8.9%, 38.4%, and 66.2% at concentrations of 25, 50, and 100 μM, respectively. In contrast, other derivatives exhibited minimal inhibitory effects on lipid accumulation. This study also high-lights the importance of key marker genes in adipocyte differentiation, such as PPAR-γ, C/EBPα, SREBP-1c, and FABP4.30 These transcription factors and proteins regulate lipid synthesis, energy homeostasis, and lipid droplet formation in mature adipocytes. Inhibition of their expression disrupts adipocyte differentiation and significantly reduces lipid accumulation. 6,7DH-4MC demonstrated concentration-dependent inhibition of these markers, with 100 μM suppressing PPAR-γ expression by 97.4% and FABP4 by 94.7%, indicating its crucial role in inhibiting lipid droplet formation during the later stages of adipocyte differentiation.

Although EGCG is widely recognized as a potent natural anti-obesity compound and therefore served as a positive control in this study, the present findings highlight the distinct significance of 6,7DH-4MC within the coumarin scaffold. EGCG, as a complex polyphenol, exerts multi-target effects that are well established; however, structurally simple 4-methylcoumarin derivatives have not previously been shown to achieve comparable inhibition of lipid accumulation or to modulate multiple adipogenic signaling pathways. The strong dose-dependent activity of 6,7DH-4MC, together with its ability to suppress key adipogenic markers at levels similar to or greater than EGCG, suggests that 6,7DH-4MC represents a chemically tractable and mechanistically novel scaffold for anti-adipogenic intervention. This distinction underscores the importance of the present study and demonstrates that coumarin derivatives may hold broader therapeutic value than previously recognized.

The anti-obesity mechanism plays a crucial role in suppressing adipocyte differentiation and lipid accumulation through the regulation of various signaling pathways. The phosphorylation of AKT regulates cell growth and survival, promoting the expression of PPAR-γ and C/EBPα, which in turn induces adipocyte differentiation. However, by inhibiting AKT phosphorylation, it is possible to discourage adipocyte differentiation and regulate lipid metabolism, thereby preventing lipid accumulation [31]. CREB is phosphorylated through the cAMP signaling pathway, activating the ex-pression of C/EBPβ, which is important in the early stages of adipocyte differentiation as it enhances the expression of PPAR-γ and C/EBPα. Although CREB plays a critical regulatory role during the early phase of adipogenesis, only the late-stage (Day 8) phosphorylation levels were evaluated in this study. Early time-point CREB activation was not assessed, and this remains a limitation that warrants further investigation.

Additionally, the MAPK signaling pathway, which includes ERK1/2 and p38 MAPK, is involved in the growth and differentiation of adipocytes, and the regulation of this pathway plays a vital role in promoting adipocyte differentiation and energy consumption. These mechanisms provide a basis for substances like 6,7DH-4MC to exhibit anti-obesity effects. Furthermore, AMPK activates in response to the cellular energy status, suppressing lipid synthesis and promoting fatty acid oxidation to exhibit anti-adipogenic effects [43,44,45,46]. Therefore, we investigated the anti-adipogenic mechanisms of 6,7DH-4MC in regulating adipocyte differentiation and lipid metabolism, examining the relationship between AKT, AMPK, and MAPK signaling pathways, and the phosphorylation of CREB through Western blot experiments. The results show that while 6,7DH-4MC had a relatively mild effect on inhibiting AKT phosphorylation in 3T3-L1 adipocytes, it effectively suppressed P-CREB, thereby inhibiting adipocyte differentiation. In the MAPK signaling pathway, 6,7DH-4MC was found to effectively inhibit the phosphorylation of ERK1/2 and p38 MAPK, regulating lipid metabolism. Importantly, it also demonstrated the effect of activating the AMPK signaling pathway, leading to the suppression of lipid synthesis and the promotion of fatty acid oxidation [47,48]. Through these multifaceted actions, 6,7DH-4MC possesses the potential to inhibit adipocyte differentiation and exert anti-obesity effects.

Although 3T3-L1 adipocytes represent a widely accepted and well-characterized in vitro model for studying adipogenesis, they do not fully recapitulate the endocrine regulation, depot-specific characteristics, or microenvironmental complexity of in vivo white adipose tissue. Therefore, the signaling responses observed in this study should be interpreted as cellular-level mechanistic insights rather than direct predictions of whole-body metabolic outcomes. To establish physiological relevance, future studies using primary adipocytes, adipose tissue explants, and in vivo models will be essential to further validate the anti-adipogenic actions of 6,7DH-4MC.

While this study demonstrates the significant anti-adipogenic effects of 6,7DH-4MC and its ability to modulate signaling pathways involved in adipocyte differentiation and lipid metabolism, certain limitations must be addressed. A key limitation is the need for additional mechanistic studies to further elucidate the interactions of 6,7DH-4MC with molecular targets such as AMPK, MAPK, and PPAR-γ. Additionally, although the in vitro results in 3T3-L1 adipocytes are promising, preclinical in vivo studies are required to validate the efficacy and safety of 6,7DH-4MC in more complex biological environments. Furthermore, clinical trials will be essential to evaluate the compound’s bioavailability, metabolism, and long-term safety in humans.

At the molecular level, 6,7-DH-4MC exhibits notable adaptability and stability in multi-target inhibition of adipogenesis. Residue energy decomposition analysis revealed that interactions in ERK1/2, PPAR-γ, and FABP4 were dominated by key aromatic residues such as TYR36, TYR327, and PHE57, whereas in p38, both aromatic and polar residues (PHE169 and GLU71) contributed substantially, and in CREB, multiple residues formed a dispersed yet stable network of aromatic interactions. Overall, these interactions were predominantly hydrophobic and aromatic, in close agreement with molecular docking predictions, further supporting the reliability of the docking conformations. Binding free energy analysis indicated that van der Waals interactions were the primary driving force stabilizing all complexes. Collectively, these results demonstrate that 6,7-DH-4MC establishes a stable multi-target binding network, predominantly mediated by hydrophobic and aromatic interactions, providing a molecular basis for its multi-level regulation of adipogenesis.

## 4. Materials and Methods

### 4.1. Chemicals and Antibodies

For this study, 4-methylcoumarin (4MC), 7-amino-4-methylcoumarin (7A-4MC), 7-methoxy-4-methylcoumarin (7M-4MC), 6,7-dimethoxy-4-methylcoumarin (6,7DM-4MC), 6,7-dihydroxy-4-methylcoumarin (6,7DH-4MC), 7,8-dihydroxy-4-methylcoumarin (7,8DH-4MC), 5,7-dihydroxy-4-methylcoumarin (5,7DH-4MC), and 6-methoxy-4-methylcoumarin (6M-4MC) were purchased from Tokyo Chemical Industry (Chuo-ku, Tokyo, Japan). The reagents used for cell culture, including Dulbecco’s Modified Eagle’s Medium (DMEM), penicillin-streptomycin (10,000 U/mL), fetal bovine serum (FBS), and bovine serum (B/S), were obtained from Thermo Fisher Scientific (Waltham, MA, USA). For protein quantification, the BCA protein assay kit was used, and hydrophilic polyvinylidene fluoride (PVDF) membrane discs for Western blotting were also purchased from Thermo Fisher Scientific. The MTT reagent (3-(4,5-dimethylthiazol-2-yl)-2,5-diphenyltetrazolium bromide) for cell viability assays, protease inhibitor cocktail and 2-mercaptoethanol for cell lysis, and reagents for adipocyte differentiation, including 3-isobutyl-1-methylxanthine, dexamethasone, insulin solution (human), and oil red O solution, were purchased from Sigma-Aldrich (St. Louis, MO, USA). The reagents for preparing Western blot samples, such as 2× Laemmli sample buff-er, 10% Tween 20, and nonionic detergent, were acquired from Bio-Rad (Hercules, CA, USA). Bovine serum albumin (BSA) was obtained from Bovostar (Bovogen, Melbourne, Australia). Dimethyl sulfoxide (DMSO), 20X TBS buffer (pH 7.6) for 1× TBST preparation, 10× Tris-glycine buffer, and phosphate-buffered saline (PBS) were obtained from Biosesang (Seongnam, Gyeonggi-do, Republic of Korea). For Western blot analysis, the β-actin antibody (C4) (C-47778_s) was purchased from Santa Cruz Biotechnology (Dallas, TX, USA). Other primary antibodies, including P-ERK (9102S), ERK (9101S), P-p38 (9211S), p38 (9212S), JNK (9252S), P-JNK (9251S), P-AKT (9271S), AKT (9272S), C/EBPα (2295S), P-CREB (9198S), PPAR-γ (2443S), FABP4 (2120S), SREBP-1 (sc-365513), as well as anti-rabbit IgG HRP-linked antibody (7074S) and anti-mouse IgG HRP-linked antibody (7076S), were obtained from Cell Signaling Technology (Danvers, MA, USA).

### 4.2. Cell Culture

For the lipid accumulation inhibition experiments, 3T3-L1 preadipocytes were obtained from the American Type Culture Collection (ATCC, Rockville, MD, USA). These cells were cultured at 37 °C with 5% CO_2_ in DMEM containing 10% bovine calf serum (B/S) and 1% penicillin-streptomycin.

### 4.3. Adipocyte Differentiation

3T3-L1 preadipocytes were seeded at a density of 7 × 10^4^ cells/well in a 12-well plate and cultured for 5 days in DMEM containing 10% bovine serum (B/S) and 1% penicillin-streptomycin. Once the cells reached confluence and adhered to the plate, adipocyte differentiation was induced on Day 0 by treating the cells with an MDI mixture consisting of 3-isobutyl-1-methylxanthine (IBMX, 0.5 mM), dexamethasone (DEX, 1 μM), and insulin (10 μg/mL). The cells were incubated for 72 h to promote adipocyte differentiation. After the initial induction period, the medium was replaced with fresh DMEM containing 10 μg/mL insulin to support the differentiation process, and the cells were incubated for an additional 3 days. Subsequently, the medium was switched to a post-differentiation medium containing DMEM, 10% FBS, and 1% penicillin-streptomycin and cultured for 2 more days (from Day 6 to Day 8). By Day 8, adipocyte differentiation was complete. During this period, various concentrations of 4-methylcoumarin derivatives were administered to evaluate their inhibitory effects on lipid accumulation during adipocyte differentiation.

### 4.4. Cell Viability

3T3-L1 preadipocytes were seeded at a density of 5.0 × 10^4^ cells/well in a 24-well plate and incubated for 24 h at 37 °C in a 5% CO_2_ incubator. After incubation, the cells were treated with various concentrations of the test samples and further incubated for 72 h under the same conditions. Following the treatment, the culture medium was removed, and 500 μL of MTT solution (0.2 mg/mL) was added to each well. The cells were incubated for an additional 4 h at 37 °C in a 5% CO_2_ atmosphere. After incubation, the MTT solution was removed, and the formazan crystals adhering to the wells were dissolved in DMSO. The plate was then incubated at 37 °C for 20 min to ensure complete dissolution. Subsequently, 200 μL of the dissolved solution from each well was transferred to a 96-well plate, and the absorbance was measured at 540 nm using a microplate reader (Epoch, BioTek, Winooski, CA, USA).

Based on the results of the MTT assay, all 4MC derivatives—including 6,7DH-4MC—maintained ≥90% viability at concentrations up to 50 μM, indicating an absence of cytotoxicity (Figure 2). Although 6,7DH-4MC showed mildly elevated viability (>110%) at 50–100 μM, no morphological abnormalities were observed. Therefore, 25 μM and 50 μM were selected as non-cytotoxic and physiologically relevant concentrations for mechanistic studies, whereas 100 μM was additionally included for 6,7DH-4MC to evaluate its full dose–response activity within the safe range.

### 4.5. Oil Red O Staining

To assess lipid accumulation in differentiated 3T3-L1 adipocytes, Oil Red O staining was performed. This method makes intracellular lipid droplets visible by staining them red, allowing for a qualitative evaluation of lipid accumulation. After differentiation was completed, the cells were washed once with 1× PBS and fixed with 10% formalin for 30 min. Following fixation, the cells were rinsed three times with distilled water. Subsequently, 500 μL of Oil Red O solution was added to each well, and the cells were stained in the dark for at least 30 min. After staining, the cells were washed three times with dis-tilled water to remove excess dye. The stained cells were then treated with 500 μL of isopropanol for 20 min with gentle shaking to extract the dye. The extracted solution was transferred to a 96-well plate, and the absorbance was measured at 520 nm using a microplate reader.

### 4.6. Western Blot Analysis

3T3-L1 preadipocytes were seeded in 60 mm culture dishes and cultured until Day 0. Afterward, the cells were treated with various concentrations of the test samples and MDI differentiation medium for specified time points to examine the expression of target proteins. Following the removal of the culture medium, the cells were washed twice with ice-cold 1× PBS. A total of 200 μL of lysis buffer was added to each dish, and the cells were incubated for 30 min at 4 °C to ensure lysis. The lysates were collected using a cell scraper and transferred to E-tubes, followed by centrifugation at 15,000 rpm for 30 min at −8 °C. The supernatants were collected, and protein concentrations were quantified using the Pierce™ BCA Protein Assay Kit (Thermo Fisher Scientific, Waltham, MA, USA). The protein samples were diluted to a uniform concentration of 30 μg/mL. A 2× Laemmli sample buffer mixed with 2-mercaptoethanol at a 20:1 ratio was combined with the protein samples at a 1:1 ratio and heated at 100 °C for 5 min to prepare the loading samples. A total of 16 μL of the prepared samples was loaded into each well of the SDS-polyacrylamide gel, followed by electrophoresis at 100 V for 30 min and 200 V for 40 min to separate the proteins based on size. The proteins were transferred onto a PVDF membrane using the Trans-Blot Turbo Transfer System. The membrane was washed four times with 1× TBS-T (prepared with 10% Tween 20, 20× TBS buffer, and dis-tilled water at a 1:5:94 ratio) for 5 min intervals. The membrane was blocked with 5% BSA (in 1× TBS-T) for 1 h and washed six times at 5 min intervals with 1× TBS-T. The primary antibodies were diluted at a 1:2000 ratio in 1× TBS-T and incubated with the membrane overnight at 4 °C. After incubation, the membrane was washed six times with 1× TBS-T at 5 min intervals. The secondary antibodies were diluted at a 1:1000 ratio in 1× TBS-T and incubated with the membrane at room temperature for 1 h and 30 min. The membrane was washed again six times with 1× TBS-T, and the protein bands were detected using an enhanced chemiluminescence (ECL) kit (Thermo Fisher Scientific, Rockford, IL, USA). The bands were visualized using the ChemiDoc Imaging System (Bio-Rad, Hercules, CA, USA), and their intensities were analyzed using ImageJ software (version 9.4.0).

### 4.7. Statistical Analyses

All data are expressed as the mean ± standard deviation (SD) of three independent experiments. Statistical significance was assessed using one-way analysis of variance (ANOVA) with WINKS SDA (v.7.9.0, TexaSoft, Cedar Hill, TX, USA). A *p*-value of <0.05 was considered statistically significant.

### 4.8. Pharmacokinetic and Drug-likeness

Pharmacokinetic properties were assessed through in silico prediction of ADME and drug-likeness using multiple computational platforms, including ADMETlab 3.0 (https://admetlab3.scbdd.com/, accessed on 20 November 2024), SwissADME (https://www.swissadme.ch/ accessed on 20 November 2025), and pkCSM (https://biosig.lab.uq.edu.au/pkcsm/; accessed on 20 November 2024).

### 4.9. Molecular Docking Analyses

The crystal structures of ERK1/2 (PDB ID: 5LCJ), p38 (PDB ID: 1A9U), CREB (PDB ID: 4WHU), PPAR-γ (PDB ID: 2PRG), and FABP4 (PDB ID: 3FR2) were obtained from the RCSB Protein Data Bank (https://www.rcsb.org/). Proteins were prepared in PyMOL v3.0.3 by removing water molecules and heteroatoms, followed by hydrogen addition. Ligands were downloaded from PubChem (https://pubchem.ncbi.nlm.nih.gov/) and energy-minimized using the MMFF94 force field in Open Babel v2.4.1. Protonation states and rotatable bonds were assigned via AutoDock Tools v1.5.6. Docking grids were defined around the co-crystallized ligand sites (15 × 15 × 15 Å, spacing 0.375 Å). To validate the docking protocol, co-crystallized ligands were re-docked into their binding pockets, and the RMSD values between pre-docking and docked poses were below 2 Å, confirming reliable docking accuracy. Docking simulations were performed using AutoDock Vina v1.2.0 in semi-flexible mode with an exhaustiveness of 25, applying the Lamarckian Genetic Algorithm. Protein–ligand interactions were analyzed with Discovery Studio 2019.

### 4.10. Molecular Dynamics Simulations Analyses

MD simulations were performed using GROMACS 2021. Proteins and ligands were parameterized with the AMBER14SB and GAFF2 force fields, respectively. Each complex was solvated in a TIP3P water box, maintaining at least 1.2 nm between the solute and box edges, and neutralized with Na^+^/Cl^−^ ions to reach a 0.15 M physiological salt concentration. Energy minimization was conducted via the steepest descent method for 50,000 steps, with a convergence threshold of 100 kJ·mol^−1^·nm^−1^. Equilibration consisted of two stages using GROMACS 2025.3 software (GROMACS Development Team, Uppsala University, Uppsala, Sweden): (i) 1 ns under the NVT ensemble using the V-rescale thermostat (310 K, τ = 0.1 ps), followed by (ii) 1 ns under the NPT ensemble with the V-rescale thermostat (310 K, τ = 0.5 ps) and the Parrinello–Rahman barostat (1 atm, τ = 1 ps). During this phase, heavy atoms of proteins and ligands were restrained with a force constant of 1000 kJ·mol^−1^·nm^−2^. Production simulations were carried out under NPT conditions without restraints, using periodic boundary conditions. Long-range electrostatics were treated with the Particle Mesh Ewald method, and a 1.2 nm cutoff was applied for short-range interactions. Hydrogen-involving bonds were constrained using LINCS, permitting a 2 fs integration step. Each system was simulated three times to ensure statistical reliability.

## 5. Conclusions

This study demonstrated that among various 4-methylcoumarin (4MC) derivatives, 6,7-dihydroxy-4-methylcoumarin (6,7DH-4MC) exhibited the most potent anti-adipogenic activity in 3T3-L1 adipocytes without cytotoxicity. It significantly reduced lipid accumulation and suppressed the expression of key adipogenic markers, including PPAR-γ, C/EBPα, SREBP-1c, and FABP4, in a concentration-dependent manner. Mechanistically, 6,7DH-4MC inhibited the phosphorylation of ERK1/2, p38 MAPK, and CREB, while activating AMPK, indicating its capacity to simultaneously regulate multiple signaling cascades that control adipocyte differentiation and lipid metabolism.

Molecular docking and dynamics simulations further supported the experimental results, revealing that 6,7DH-4MC forms stable hydrophobic and hydrogen-bonding interactions within the active sites of PPAR-γ, FABP4, ERK1/2, p38, and CREB. These interactions highlight a multi-target binding mechanism that underlies its pleiotropic anti-adipogenic effects.

Collectively, these findings suggest that 6,7DH-4MC is a promising natural coumarin-derived scaffold capable of modulating multiple signaling pathways associated with adipogenesis. Its dual action—inhibition of MAPK-mediated adipogenic signaling and activation of AMPK-mediated lipid catabolism—positions it as a potential lead compound for developing novel and safer anti-obesity therapeutics. Future in vivo and clinical studies will be essential to validate its pharmacological efficacy, bioavailability, and safety profiles.

## Figures and Tables

**Table 1:** ADMET properties of the compounds.

ADMET Properties		6,7DH-4MC	EGCG
Absorption			
Caco-2 permeability (cm/s) ^a^		−4.63	−0.66
P-gp protein inhibitor ^a^		No	Yes
P-gp substrate ^a^		Yes	Yes
Human intestinal absorption ^a^		93.81%	57.66%
Distribution			
Plasma protein binding ^b^		72.1%	87.3%
Volume distribution (L/kg) ^a^		−0.532	−1.516
Blood–brain barrier ^a^		−0.009	−1.786
Metabolism			
CYP450	CYP1A2 inhibitor ^c^	Yes	No
	CYP2C19 inhibitor ^c^	No	No
	CYP2C9 inhibitor ^c^	No	No
	CYP2D6 inhibitor ^c^	No	No
	CYP3A4 inhibitor ^c^	No	Yes
Elimination			
Clearance rate (mL/min/kg) ^a^		0.67	0.36
T_1/2_ (h) ^b^		1.662	−0.11

T_1/2_: time required for the plasma concentration of a drug to decrease by 50%; ^a^: pkCSM; ^b^: ADMETlab 3.0; ^c^: SwissADME.

**Table 2:** Drug-likeness properties of compounds.

Compound	MW ^a^	HBA ^a^	HBD ^a^	RB ^a^	TPSA ^a^	Log *p* ^a^	MR ^b^	RO5 ^b^	Ghose Filter ^b^	Veber Rule ^b^	Egan Rule ^b^
6,7DH-4MC	192.0	4	2	0	70.7	1.1	51.5	Yes	Yes	Yes	Yes
EGCG	458.4	11	8	3	197.4	2.2	112.0	No *	Yes	No **	No ***

MW: molecular weight, (g/mol); HBA: number of H-Bond acceptors; HBD: number of H-Bond donors; RB: number of rotatable bonds; TPSA: Topological Polar Surface Area, (Å^2^); MR: molar refractivity; *: 2 violations: N or O > 10, NH or OH > 5; **: 1 violation: TPSA > 140; ***: 1 violation: TPSA > 131.6. ^a^: ADMETlab 3.0; ^b^: SwissADME.

**Table 3:** Docking results of 6,7-DH-4MC with target proteins.

Complex	Docking Energy	Hydrophilic Interactions	Hydrophobic Interactions
6,7DH-4MC–ERK1/2	−6.5 kcal/mol	ASP106, MET108	ILE31, VAL39, ALA52, LEU156
6,7DH-4MC–p38	−6.6 kcal/mol	LYS53	GLU71, LEU75, ASP168, PHE169
6,7DH-4MC–CREB	−6.8 kcal/mol		PHE1111, VAL1115, LEU1120, ILE1122, ALA1164, TYR1167, ASN1168, VAL1174
6,7DH-4MC–PPAR-γ	−6.4 kcal/mol	TYR327	ARG288, CYS285, ILE326, TYR327, LEU330, MET364
6,7DH-4MC–FABP4	−6.4 kcal/mol	ASP76, ARG126, TYR128	PHE16, ALA33, PHE57, ALA75

**Table 4:** MM/GBSA Energy Components of 6,7DH-4MC–Protein Complexes. (vdW, Van der Waals energy; EEL, Electrostatic energy; EGB, Polar solvation energy; ESURF, Nonpolar solvation energy; GGAS, Gas-phase energy; GSOLV, Solvation energy; kcal/mol).

Complex	vdW	EEL	EGB	ESURF	GGAS	GSOLV	Total Binding Energy
6,7DH-4MC–ERK1/2	−30.59	−9.36	20.44	−3.53	−39.96	16.91	−23.05
6,7DH-4MC–p38	−25.03	−7.04	17.69	−3.52	−32.07	14.17	−17.90
6,7DH-4MC–CREB	−23.51	−7.10	13.61	−3.46	−30.61	10.15	−20.45
6,7DH-4MC–PPAR-γ	−27.84	−9.11	17.85	−3.90	−36.95	13.95	−23.00
6,7DH-4MC–FABP4	−21.61	−2.60	10.94	−2.89	−24.20	8.05	−16.15

**Table 5:** Residue Energy Decomposition of 6,7DH-4MC–Protein Complexes.

Complex	Key Residues	ΔG_residue_ (kcal/mol)
6,7DH-4MC–ERK1/2	TYR36, GLY37, MET38, ALA52, GLU71	−2.72, −0.94, −0.95, −1.90, −0.61
6,7DH-4MC–p38	GLU71, LEU74, LEU75, PHE169, ALA172	−1.76, −1.28, −1.01, −1.86, −1.09
6,7DH-4MC–CREB	PRO1110, PHE1111, VAL1115, LEU1120, ILE1122, TYR1125, ALA1164, TYR1167, ASN1168, VAL1174	−0.74, −1.51, −1.08, −0.69, −0.80, −1.01, −0.61, −0.83, −0.93, −1.01
6,7DH-4MC–PPAR-γ	CYS285, SER289, ILE326, TYR327	−1.32, −1.01, −1.13, −1.50
6,7DH-4MC–FABP4	ALA33, PRO38, PHE57	−0.97, −0.87, −3.12

## Data Availability

All data generated or analyzed during this study are fully available within this published article.
